# Maternal TLR4 and NOD2 Gene Variants, Pro-Inflammatory Phenotype and Susceptibility to Early-Onset Preeclampsia and HELLP Syndrome

**DOI:** 10.1371/journal.pone.0001865

**Published:** 2008-04-02

**Authors:** Bas B. van Rijn, Arie Franx, Eric A. P. Steegers, Christianne J. M. de Groot, Rogier M. Bertina, Gerard Pasterkamp, Hieronymus A. M. Voorbij, Hein W. Bruinse, Mark Roest

**Affiliations:** 1 Division of Perinatology and Gynecology, University Medical Center Utrecht, Utrecht, The Netherlands; 2 Laboratory for Clinical Chemistry and Hematology, University Medical Center Utrecht, Utrecht, The Netherlands; 3 Department of Experimental Cardiology, University Medical Center Utrecht, Utrecht, The Netherlands; 4 Department of Obstetrics and Gynecology, St Elisabeth Hospital Tilburg, Tilburg, The Netherlands; 5 Division of Obstetrics and Prenatal Medicine, Erasmus Medical Center Rotterdam, Rotterdam, The Netherlands; 6 Department of Obstetrics and Gynecology, Medical Center Haaglanden, The Hague, The Netherlands; 7 Department of Hematology, Leiden University Medical Center, Leiden, The Netherlands; University of Utah, United States of America

## Abstract

**Background:**

Altered maternal inflammatory responses play a role in the development of preeclampsia and the hemolysis, elevated liver enzymes and low platelets (HELLP) syndrome. We examined whether allelic variants of the innate immune receptors Toll-like receptor 4 (TLR4) and nucleotide-binding oligomerization domain 2 (NOD2), that impair the inflammatory response to endotoxin, are related to preeclampsia and HELLP syndrome.

**Methods and Findings:**

We determined five common mutations in TLR4 (D299G and T399I) and NOD2 (R702W, G908R and L1007fs) in 340 primiparous women with a history of early-onset preeclampsia, of whom 177 women developed HELLP syndrome and in 113 women with a history of only uneventful pregnancies as controls. In addition, we assessed plasma levels of pro-inflammatory biomarkers C-reactive protein, interleukin-6, soluble intercellular adhesion molecule-1, fibrinogen and von Willebrand factor in a subset of 214 women included at least six months after delivery. After adjustment for maternal age and chronic hypertension, attenuating allelic variants of TLR4 were more common in women with a history of early-onset preeclampsia than in controls (OR 2.9 [95% CI 1.2–6.7]). Highest frequencies for TLR4 variants were observed in women who developed HELLP syndrome (adjusted OR 4.1 [95% CI 1.7–9.8]). In addition, high levels of interleukin-6 and fibrinogen were associated with a history of early-onset preeclampsia. Combined positivity for any of the TLR4 and NOD2 allelic variants and high levels of interleukin-6 was 6.9-fold more common in women with a history of early-onset preeclampsia (95% CI 2.1–23.2) compared to controls.

**Conclusions:**

We observed an association of common TLR4 and NOD2 gene variants, and pro-inflammatory phenotype with a history of early-onset preeclampsia and HELLP syndrome. These findings suggest involvement of the maternal innate immune system in severe hypertensive disorders of pregnancy.

## Introduction

Preeclampsia is a complex multi-system disease of unknown origin, characterized by hypertension and proteinuria in the second half of pregnancy, with potentially life-threatening consequences for both mother and baby [1]. Worldwide an estimated 4.2 million women are affected by preeclampsia each year and an annual 60,000 maternal deaths are attributed to the disorder [2].

The inflammatory system has a pivotal role in the pathogenesis of common placental and hypertensive disorders of pregnancy, including preeclampsia, intra-uterine growth restriction (IUGR) and the hemolysis, elevated liver enzymes and low platelets (HELLP) syndrome [1], [3], [4]. Maternal adaptation to pregnancy requires a prominent and tightly regulated interplay of innate and adaptive immunity to allow normal growth and development of the semi-allogeneic fetus. Inappropriate inflammatory patterns have been related to abnormal trophoblast invasion [5], endothelial damage [3], and renal dysfunction [6]. Furthermore, preeclampsia is characterized by excessive production of pro-inflammatory cytokines and chemokines [7], as well as disturbances in immunosuppressive regulators such as IL-10 and TGF-β [7], [8]. Endotoxin or lipopolysaccharide (LPS) is a potent antigen normally present in gram-negative bacteria, known to induce a systemic pro-inflammatory reaction. LPS-mediated inflammation is believed to be of critical importance in many systemic infectious and non-infectious or auto-immune disorders, including sepsis [9], allergic asthma [10] and inflammatory bowel diseases [11]. In an animal model, Faas and colleagues demonstrated that infusion of low-dose LPS induces a preeclampsia-like syndrome, including hypertension, proteinuria and glomerular endotheliosis [12]. LPS leads to release of pro-inflammatory cytokines through activation of two major pattern recognition receptors (PRRs) present on innate immune cells (macrophages, NK cells and dendritic cells), i.e. the extracellular Toll-like receptor 4 (TLR4), and the intracellular nucleotide oligomerization domain 2 (NOD2) protein, also known as the caspase-activating recruitment domain 15 (CARD15). Upon activation, these receptors signal cells of the adaptive immune system (mainly T cells) via production of nuclear factor kappa B and release of pro-inflammatory cytokines (IL-1β, IL-6, TNF-α) to constitute an inflammatory response necessary for effective clearance of the harmful pathogen. Allelic variants of TLR4 and NOD2 have been associated with an attenuated immune response to LPS, leading to inappropriate inflammatory patterns which can be both ineffective in dealing with an infectious agent, as well as cause the adverse effects of uncontrolled inflammation [13], [14]. Genetic variation in the extracellular (TLR4) and intracellular (NOD2) endotoxin sensing system have been related to sepsis [9], Crohn's disease [11], [15], premature delivery [16] and atherogenesis [17]. In this study we examined the contribution of two common allelic variants of the TLR4 (D299G and T399I) and three common allelic variants of NOD2 (R702W, G908R and L1007fs) to the pathogenesis of early-onset preeclampsia and HELLP syndrome. In addition, we determined plasma levels of biomarkers representative for the acute-phase inflammatory response (C-reactive protein, fibrinogen, interleukin-6, soluble intercellular adhesion molecule-1 and von Willebrand factor) in women with a history of early-onset preeclampsia and controls with only uneventful pregnancies at least six months after delivery. We hypothesized that allelic variants of TLR4 and NOD2, as well as high circulating levels of pro-inflammatory biomarkers after delivery would relate to a history of early-onset preeclampsia and HELLP syndrome.

## Methods

### Participants

Participants were recruited between August 1995 and September 2005 in four tertiary referral centres (University Medical Center Utrecht; N = 280, Leiden University Medical Center; N = 45, St Radboud University Medical Center Nijmegen; N = 98 and Maxima Medical Center Veldhoven; N = 30) in the Netherlands. Inclusion criteria for cases were primiparity, a history of preeclampsia or HELLP syndrome, and delivery before 34 weeks of gestation. Controls were women with a history of at least one uneventful pregnancy, recruited at the University Medical Center Utrecht (N = 75) and St Radboud University Medical Center Nijmegen (N = 38). At inclusion, demographic data and whole blood samples for DNA isolation were obtained from all participants. For a subgroup of 214 women (all recruited at the University Medical Center Utrecht, at least six months after delivery; N = 144 cases), plasma samples were available for analysis of metabolic, lipid and inflammatory markers. Inclusion criteria, sample collection and data acquisition were previously described elsewhere [18]–[20]. The study was approved by the Medical Ethics Committee of the University Medical Center Utrecht, the Medical Ethics Committee of the University Medical Center Nijmegen, the Institutional Review Board of the Maxima Medical Center Veldhoven and the Medical Ethics Committee of the Leiden University Medical Center, and written informed consent was obtained from all participants. Preeclampsia was defined according to the criteria of the International Society for the Study of Hypertension in Pregnancy [21], as de novo hypertension above 90 mm Hg at two or more consecutive measurements, at least 4 hours apart, and proteinuria above 0.3 g per 24 hours. HELLP syndrome was defined according to previously described criteria [22], as hemolysis (defined as serum lactate dehydrogenase (LDH) >600 U/L and/or haptoglobin ≤0.3 g/L), elevated liver enzymes (serum aspartate aminotransferase (AST) >70 U/L and/or serum alanine aminotransferase (ALT) >70 U/L), and a low platelet count (<100×10^9^/L). Small-for-gestational age (SGA) was defined as birth weight below the 5th centile, based on the Dutch population charts [23]. Finally, chronic hypertension was defined as hypertension, treated with anti-hypertensive medication initiated before first pregnancy.

### Procedures

Genomic DNA was isolated from blood, using standard commercially available kits (Gentra Systems, Minneapolis, MN, USA). Two common missense mutations of the TLR4 gene (GenBank: NM_138554; OMIM: 603030) were detected by a previously described polymerase chain reaction (PCR) [24], based on exonuclease degradation of dual labelled allele-specific oligonucleotides: a substitution of adenosine by guanine at position 896 in the fourth exon, leading to replacement of aspartic acid by glycine at amino acid position 299 in the extracellular domain of TLR4 (D299G; RefSNP ID: rs4986790) and a frequently co-segregating mutation, leading to an amino acid substitution at position 399 of threonine by isoleucine (T399I; RefSNP ID: rs4986791). In addition, three common allelic variants of the NOD2 gene (GenBank: NM_022162; OMIM: 605956) were determined by a similar genotyping method: a substitution of cytosine by thymine at position 2104 leading to amino acid replacement of arginine by tryptophan at position 702 in the fourth exon (R702W; RefSNP ID: rs2066844), a substitution of cytosine by guanine at position 2722 leading to replacement of glycine at arginine at position 908 in the eighth exon (G908R; RefSNP ID: rs2066845) and an insertion of cytosine at position 3020 leading to an amino acid frame shift from leucine at position 1007 (L1007fs; RefSNP ID: rs2066847). Primers and probes were designed by prevalidated commercially available custom-made development kits (Assay-by-Design; Applied Biosystems, Foster City, CA, USA) and performed on a 384-wells PCR machine (Biometra, Goettingen, Germany) and an automated fluorimeter (Fluostar Galaxy, BMG LABTECH, Offenburg, Germany), as described in detail before [24]. Genotyping assays were performed by operators blinded to phenotypic outcome, replicated in a randomly selected sample set and included control samples for each of the genotypes, verified by DNA sequencing.

For phenotype analysis, fasting blood samples were collected, immediately centrifuged and plasma was stored at −80°C until further analysis. Plasma concentrations of high sensitivity C-reactive protein (CRP) were determined using a commercially available reagents for nephelometric analysis (Dade Behring, Marburg, Germany). Fibrinogen levels were measured by the Claus' clotting method using the Sta-R automatic coagulation analyzer with STA Fibrinogen reagent (Diagnostic Stago, Taverny, France). Plasma levels of interleukin-6 (IL-6) and soluble intercellular adhesion molecule-1 (sICAM-1) were assessed by means of commercially available enzyme-linked immunosorbent assay development kits (R&D Systems, Minneapolis, MN, USA), according to the manufacturer's protocol. Plasma antigen levels of von Willebrand factor (vWf) were measured with an enzyme-linked immunosorbent assay using commercial antibodies (Dako, Glostrup, Denmark), as previously described [25]. Intra-assay and inter-assay coefficients of variability were <10% for all assays. Lower detection limit for C-reactive protein was 0.15 mg/L, with a coefficient of variability of 3.3%. The fibrinogen assay had a lower detection limit of 0.28 g/L, with a coefficient of variability of 4.1%. For the commercial ELISA kits to measure IL-6, the lower detection limit in plasma was 1.0 pg/mL with a coefficient of variability of 7.2%, and to measure sICAM, the lower limit was 31 ng/mL with a coefficient of variability of 7.1%. Finally, von Willebrand factor assay had a lower detection limit of 1.2 µg/mL with a coefficient of variability of 8.0%.

### Statistics

Baseline and clinical outcome variables were compared by Students t-tests for continuous variables and Chi-squared tests for categorical variables where appropriate. The relative contributions of TLR4 and NOD2 genotypes to the occurrence of early-onset preeclampsia were estimated by logistic regression analysis, and expressed as odds ratios with 95% confidence intervals (CIs). Because time of diagnosis and the presence of pre-existent hypertensive disorders could theoretically influence the diagnosis of preeclampsia, odds ratios were adjusted for age at first delivery and chronic hypertension, by multivariable logistic regression analysis. To account for their skewed distributions, circulating levels of inflammatory biomarkers were presented as medians with interquartile range, and comparisons between cases and controls were based on Mann-Whitney *U* statistics. Correlations between continuous variables were calculated with the use of age-adjusted Spearman partial correlation coefficients. In addition, interactions between inflammatory markers and allelic variants of TLR4 and NOD2 were evaluated by calculating odds ratios for occurrence of preeclampsia or HELLP syndrome, according to each tertile of the plasma level distribution of the control population, also by logistic regression analysis. To evaluate the combined contributions of genotype and tertiles of plasma inflammatory markers, trend analysis was performed by logistic regression analysis among the following groups using a regression equation for early-onset preeclampsia [26]: women negative for any of the minor allelic variants of TLR4 and NOD2 and within the lowest tertile of plasma inflammatory markers, according to the distribution among controls (reference group; score = 1), women negative for allelic variants within the highest tertiles and women positive for one or more allelic variants within the lower two tertiles (score = 2), and women positive for one or more allelic variants within the highest tertiles (score = 3). Subsequently, multivariable logistic regression analysis was used to control for age at inclusion, differences in body-mass index (less than 20, 20 to 25, 25 to 30 and 30 or more), interval between delivery and inclusion, current smoker (yes or no), and chronic hypertension (yes or no).

## Results

Women with a history of early-onset preeclampsia had significantly higher body-mass index and slightly lower age at enrolment in the study than controls. Among the 340 women with first pregnancy early-onset preeclampsia, 177 women developed HELLP syndrome, and 98 delivered small-for-gestational age infants (Table 1).

**Table 1:** Baseline Characteristics, First Pregnancy Outcome and Plasma Markers of Inflammation and the Acute-Phase Response

Characteristic	Early-Onset Preeclampsia (N = 340)	Controls (N = 113)	P Value
Maternal age at inclusion – yr	30.6±4.4	32.8±4.3	<0.001
Maternal age at delivery – yr	29.7±4.4	31.1±4.2	0.005
Height – cm	169±7	169±10	0.73
Weight – kg	73±15	66±11	0.001
Body-mass index	25.7±5.3	22.7±4.0	<0.001
Chronic hypertension – no. (%)	61 (19)	7 (7)	<0.001
Ethnicity – caucasian no. (%)	326 (97)	112 (99)	0.21
Current smoker – no. (%)	43 (17)	19 (21)	0.31
Smoking during pregnancy † – no. (%)	59 (18)	16 (18)	0.92
Gestational age at delivery † – wk	29.9±2.4	40.0±1.3	<0.001
Infant's birth weight † – g	1090±427	3576±469	<0.001
Hellp-syndrome † – no. (%)	177 (51)	-	-
Small-for-gestational-age infant (<5th centile) † – no. (%)	98 (28)	1 (1)	<0.001
C-reactive protein – mg/L	0.9±2.8	0.6±1.0	0.05
Fibrinogen – g/L	2.8±1.2	2.5±1.4	0.02
Interleukin-6 – pg/mL	3.6±2.7	2.8±2.4	0.002
sICAM-1 – ng/mL	121±148	138±121	0.04
von Willebrand factor – µg/mL	8.4±5.0	8.2±3.6	0.52

Data are expressed as means±SD, or as number (%) and compared by the independent sample T-test. Plasma inflammatory markers were measured in a subset of 214 women at least six months after delivery and are expressed as medians±interquartile range and compared by the nonparametric Mann-Whitney *U* test.

† Data represent outcomes of first pregnancy.

All genotype frequencies were in Hardy-Weinberg equilibrium with allele frequencies for TLR4 at 7.4% for the D-allele at position 299 and 7.2% for the I-allele at position 399, and for NOD2 at 4.4% for the W-allele at position 702, 1.0% for the R-allele at position 908 and 1.8% for the frame shift deletion at position 1007. As expected, TLR4 D299G and T399I genotypes showed a high co-segregation rate of 97%.

**Figure 1:**
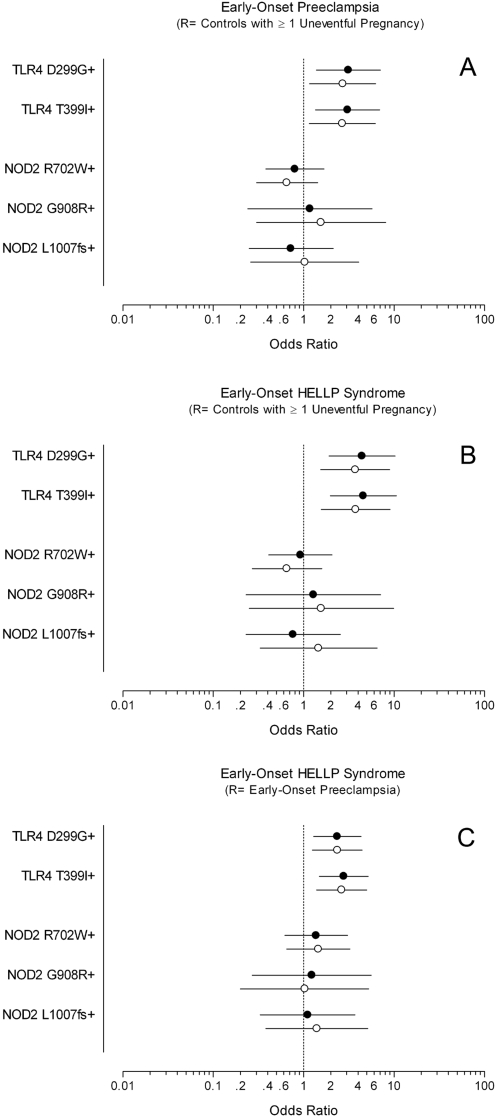
Association of Early-Onset Preeclampsia and HELLP Syndrome with Common Allelic Variants of TLR4 and NOD2 Related to Impaired Innate Inflammatory Responses to Endotoxin. Odds ratios (95% confidence intervals) for carrying either heterozygous or homozygous common functional allelic variants of TLR4 (D299G and T399I) and NOD2 (R702W, G908R and L1007fs) are presented as unadjusted values (closed symbols) and were adjusted for maternal age at first delivery and chronic hypertension (open symbols). Comparisons were made between women with a history of early-onset preeclampsia and controls with a history of only uneventful pregnancies (A), between women with a history of early-onset HELLP syndrome and controls with a history of only uneventful pregnancies (B) and between women with a history of early-onset HELLP syndrome and women with a history of early-onset preeclampsia without HELLP syndrome (C).

Among all women with a history of early-onset preeclampsia, positivity for one or more of the minor allelic variants of TLR4 was observed significantly more often than in controls, with an odds ratio of 3.3 (95% CI 1.5 to 7.5). This association between TLR4 genotype and outcome remained significant (adjusted OR 2.9 [95% CI 1.2 to 6.7]), after adjustment for maternal age and chronic hypertension in our multivariable model (Figure 1a). In women with early-onset preeclampsia complicated by HELLP syndrome (N = 177), positivity for one or more allelic variants of TLR4 was observed in 1 out of 4 women, with an crude odds ratio of 4.7 (95% CI 2.0 to 10.9) and adjusted odds ratio of 4.1 (95% CI 1.7 to 9.8), when compared to controls with only uneventful pregnancies (Figure 1b). In addition, TLR4 polymorphisms were significantly more common in women who developed HELLP syndrome, when compared to women with preeclampsia only (excluding those with HELLP) with an OR of 2.3 (95% CI 1.3 to 4.3) after adjustment for maternal age and chronic hypertension (Figure 1c). Individually, each of the NOD2 allelic variants (R702W, G908R and L1007fs) did not contribute to the development of early onset preeclampsia with or without HELLP syndrome (Figure 1).

Median levels of CRP, fibrinogen, and IL-6 were significantly higher in women with a history of early-onset preeclampsia (Table 1). Conversely, median sICAM-1 levels were lower in cases and vWf levels showed no difference between cases and controls. We observed no difference in median plasma levels of inflammatory markers between women with a history of HELLP syndrome, compared to women with early-onset preeclampsia without HELLP syndrome (data not shown).

Correlations between plasma levels of inflammatory markers (CRP, IL-6, sICAM-1), acute-phase reactants (fibrinogen, vWf) and baseline variables are shown in Table 2. In women with a history of early-onset preeclampsia significant positive correlations were observed between CRP, sICAM-1, fibrinogen and vWf, ranging from 0.20 for CRP and vWf to 0.38 for fibrinogen and vWf and for CRP and fibrinogen. In addition, body-mass index related to plasma levels of fibrinogen in cases (0.30) and CRP in controls (0.32). Interestingly, plasma levels of CRP, fibrinogen and vWf were unaffected by the time between delivery and enrolment for both cases and controls. However, IL-6 levels showed a significant negative correlation with interval between delivery and inclusion for controls only (−0.33). This correlation was not observed within women with a history of early-onset preeclampsia, probably due to the significantly higher median IL-6 levels in women with a history of early-onset preeclampsia (3.6±2.7 pg/mL compared to 2.8±2.4 pg/mL; p = 0.002). A similar effect of interval between delivery and inclusion upon sICAM-1 levels (−0.28; p<0.001) was observed for cases only.

**Table 2:** Age-Adjusted Spearman Partial Correlation Coefficients between Plasma Inflammatory Biomarkers, Body-Mass Index, and Interval Between Delivery and Enrolment among 70 Controls with a History of Only Uneventful Pregnancies and 144 Cases with a History of Early-Onset Preeclampsia *

Controls							
	CRP	Fibrinogen	IL-6	sICAM	vWf	BMI	Interval
CRP	–						
Fibrinogen	0.17	–					
IL-6	−0.21	0.10	–				
sICAM	0.06	0.01	0.02	–			
vWf	−0.04	−0.03	−0.06	−0.05	–		
BMI	0.22	0.32†	−0.06	−0.14	0.04	–	
Interval	0.00	−0.22	−0.33§	−0.09	−0.08	−0.07	–

Cases							
	CRP	Fibrinogen	IL-6	sICAM	vWf	BMI	Interval
CRP	–						
Fibrinogen	0.38¶	–					
IL-6	0.14	0.12	–				
sICAM	0.22§	0.13	−0.03	–			
vWf	0.20†	0.38¶	0.11	−0.03	–		
BMI	0.30§	0.12	0.05	0.08	−0.05	–	
Interval	−0.04	−0.03	0.03	−0.28‡	0.13	−0.07	–

* CRP denotes C-reactive protein, IL-6 interleukin-6, sICAM soluble intercellular adhesion molecule-1, vWf von Willebrand factor, BMI body-mass index and Interval time between delivery and enrolment.

† P value<0.05

§ P value<0.01

‡ P value<0.001

¶ P value<0.0001

Table 3 shows the relationship between plasma inflammatory markers measured at least six months after delivery and history of early-onset preeclampsia, according to tertiles based on the distribution of plasma levels within the control group of women with a history of at least one uneventful pregnancy. Higher circulating IL-6 levels within both the mid and highest tertiles were observed more frequently in women with a history of early-onset preeclampsia compared to controls (P for trend <0.001), with an unadjusted OR of 3.4 for the mid tertile (95% CI 1.5 to 7.7) and 4.9 for the highest tertile (95% CI 2.2 to 11.0). Adjustment for interval between delivery and enrolment, age at inclusion, body-mass index, chronic hypertension and smoking did not affect this relationship. In addition, a history of early-onset preeclampsia was related to lower plasma levels of sICAM-1. After adjustment for confounding variables, ORs (95% CI) for early-onset preeclampsia were 0.3 (0.1 to 0.9; P for trend 0.02) for both the mid and highest tertiles of sICAM-1.

**Table 3:** Odds Ratios (95 Percent Confidence Intervals) for Women with a History of Early-Onset Preeclampsia, Compared to Controls with Only Uneventful Pregnancies, According to Plasma Levels of Acute-Phase Inflammatory Biomarkers At Least Six Months After Delivery

	Unadjusted OR (95% CI)				Adjusted OR (95% CI) Model 1 †				Adjusted OR (95% CI) Model 2 §			
	Low (R)	Mid	High	P for trend *	Low (R)	Mid	High	P for trend *	Low (R)	Mid	High	P for trend *
CRP, mg/L [0.4–1.0]	1	0.7 (0.4–1.5)	1.6 (0.8–3.2)	0.16	1	0.8 (0.4–1.6)	1.6 (0.8–3.2)	0.17	1	0.4 (0.1–1.2)	0.9 (0.3–2.3)	0.86
Fibrinogen, g/L [2.1–2.7]	1	0.9 (0.4–2.0)	2.1 (1.0–4.5)	0.03	1	0.8 (0.4–1.8)	1.9 (0.9–4.1)	0.07	1	1.6 (0.5–5.2)	1.2 (0.4–3.4)	0.71
IL-6, pg/mL [2.1–3.5]	1	3.4 (1.5–7.7)	4.9 (2.2–11)	<0.001	1	2.9 (1.3–6.7)	3.9 (1.7–9.1)	0.002	1	3.6 (1.1–11)	4.6 (1.4–14)	0.01
sICAM-1, ng/mL [93–179]	1	0.4 (0.2–0.8)	0.6 (0.3–1.3)	0.09	1	0.3 (0.2–0.7)	0.5 (0.3–1.0)	0.06	1	0.3 (0.1–0.9)	0.3 (0.1–0.9)	0.02
vWf, µg/mL [7.1–9.4]	1	0.5 (0.3–1.1)	1.0 (0.5–1.9)	0.82	1	0.5 (0.2–1.1)	1.0 (0.5–2.0)	0.91	1	0.4 (0.2–1.2)	0.8 (0.3–1.9)	0.41

* P values represent trends accross increasing tertiles of plasma levels, by logistic regression analysis.

† Model 1 includes tertiles of plasma levels and interval between delivery and enrolment.

§ Model 2 includes tertiles of plasma levels, interval between delivery and enrolment, age at inclusion, body-mass index, current smoking and chronic hypertension.

Median levels of CRP, IL-6, sICAM-1, fibrinogen and sICAM-1 showed no direct association with heterozygous, homozygous or combined positivity for any of the TLR4 and NOD2 allelic variants (all P values >0.05 after group comparison by Kruskal-Wallis testing; data not shown). Nonetheless, increasing tertiles of interleukin-6 and fibrinogen levels were associated with higher odds ratios for early-onset preeclampsia in women carrying one or more TLR4 or NOD2 mutations, compared to women with the wild-type TLR4 or NOD2 genotype (Table 4). Overall, highest odds ratios for early-onset preeclampsia were observed for women carrying any of the five allelic variants of TLR4 or NOD2, within the highest tertiles for IL-6 and fibrinogen, with ORs of 6.9 (95% CI 2.1 to 23.2; P for trend 0.03 after adjustment for confounding variables) for IL-6 and 3.8 (95% CI 1.2 to 11.8; adjusted P for trend 0.04) for fibrinogen, respectively.

**Table 4:** Genotype-Phenotype Interactions for TLR4 and NOD2 Genotypes and Plasma Inflammatory Markers, among Women with a History of Early-Onset Preeclampsia and Controls with Only Uneventful Pregnancies *

	TLR4 negative (n = 194)		TLR4 positive (n = 36)			
	Low (R)	High	Low	High	*P Value* †	*P_a_ Value* ¶
CRP	1	1.6 (0.8–3.4)	3.9 (0.8–19.0)	3.0 (0.8–11.6)	0.05	0.13
Fibrinogen	1	2.2 (1.0–4.8)	3.5 (0.7–18.0)	4.7 (1.2–18.0)	0.007	0.02
IL-6	1	4.4 (1.9–10.3)	9.0 (1.7–46.5)	7.5 (1.8–30.8)	<0.001	0.006
sICAM	1	0.6 (0.3–1.3)	2.4 (0.7–9.0)	1.3 (0.2–6.7)	0.86	0.34
vWf	1	0.9 (0.5–1.9)	2.7 (0.7–10.1)	2.0 (0.4–10.1)	0.43	0.43

	NOD2 negative (n = 200)		NOD2 positive (n = 30)			
	Low (R)	High	Low	High	*P Value* †	*P_a_ Value* ¶
CRP	1	1.5 (0.7–3.1)	0.9 (0.3–2.8)	2.7 (0.5–13.4)	0.19	0.39
Fibrinogen	1	1.9 (0.9–4.2)	0.8 (0.2–2.6)	3.9 (0.8–19.3)	0.06	0.14
IL-6	1	4.5 (1.9–10.7)	2.1 (0.6–7.3)	9.2 (1.8–48.2)	<0.001	0.01
sICAM	1	0.6 (0.3–1.3)	1.0 (0.3–3.6)	0.7 (0.2–2.5)	0.34	0.10
vWf	1	0.8 (0.4–1.7)	0.7 (0.2–2.1)	2.1 (0.4–10.2)	0.85	0.93

	TLR4 or NOD2 negative (n = 170)		TLR4 or NOD2 positive (n = 60)			
	Low (R)	High	Low (R)	High	*P Value* †	*P_a_ Value* ¶
CRP	1	1.6 (0.7–3.5)	1.7 (0.6–4.5)	2.6 (0.8–8.0)	0.07	0.23
Fibrinogen	1	2.2 (1.0–5.1)	1.6 (0.5–4.5)	3.8 (1.2–11.8)	0.02	0.04
IL-6	1	4.3 (1.7–10.5)	3.6 (1.2–10.7)	6.9 (2.1–23.2)	<0.001	0.03
sICAM	1	0.7 (0.3–1.5)	1.6 (0.6–4.2)	0.8 (0.3–2.5)	0.76	0.27
vWf	1	0.9 (0.4–1.9)	1.3 (0.5–3.2)	1.9 (0.6–6.5)	0.43	0.57

* Data represent odds ratios and 95% CIs for early-onset preeclampsia compared to controls with at least one uneventful pregnancy, with respect to the reference group (R) of women negative for the all minor allelic variants of TLR4 and NOD2 and with low values for plasma inflammatory markers. High values are values within the highest tertiles of the distribution of plasma levels of the control group. Low values are values within the lowest tertiles (reference group) and values within the lower two tertiles (TLR4 and NOD2 positive group), respectively; CRP denotes C-reactive protein, IL-6 interleukin-6, sICAM soluble intercellular adhesion molecule-1 and vWf von Willebrand factor.

† P values for trend between groups, compared to the reference group (R) of women negative for allelic variants of TLR4 and NOD2, and with low levels of plasma inflammatory markers, by univariable logistic regression analysis.

¶ P_a_ values for trend between groups, compared to the reference group (R), after adjustment for age at inclusion, interval between delivery and enrolment, body-mass index, smoking and chronic hypertension, by multivariable logistic regression analysis.

## Discussion

We observed two novel findings from our study. First, that maternal predisposition to early-onset preeclampsia and HELLP syndrome is associated with allelic variants of genes that impair the innate immune response, as demonstrated by the association with common TLR4 polymorphisms. Second, that high circulating levels of biomarkers representative of the inflammatory response are associated with a history of early-onset preeclampsia and HELLP syndrome in apparently healthy non-pregnant women. Our findings are consistent with a central role for the maternal innate immune system in the development of severe hypertensive disorders of pregnancy. Moreover, our findings provide the first evidence for a potential role of endotoxin responsiveness in the susceptibility to preeclampsia and HELLP in humans.

Endotoxin, also known as lipopolysaccharide (LPS), has been shown to induce pregnancy-specific inflammatory and endothelial cell disturbances in rats, including hypertension and proteinuria and characteristic glomerular endotheliosis lesions, with striking similarities to the clinical syndrome of preeclampsia [12]. Taken together with human data showing elevated circulating biomarkers of soluble and cellular components of the innate immune system before, during and after pregnancies complicated by early-onset preeclampsia [3], [7], [27], we hypothesized that genetically encoded individual differences in the inflammatory response to LPS could influence maternal predisposition to the disorder. In the present study, we demonstrated that the allelic variants D299G and T399I of the extracellular pattern-recognition receptor TLR4, that impair the inflammatory response to LPS, are related to a history of early-onset preeclampsia. Alternately, we found no association for three attenuating polymorphisms (R702W, G908R and L1007fs) of the innate immune receptor NOD2, which binds the bacterial component muramyl dipeptide (MDP) and is involved in intracellular LPS-signalling [14].

Toll-like receptors (TLR) are considered as the most important class of pattern-recognition receptors (PRRs), involved in host defense against a variety of microbes by induction and regulation of innate immunity [28]. At present 10 members of the TLR family have been discovered in humans, of which TLR4 was first recognized as a key receptor for the LPS component of Gram-negative bacteria [13]. In addition, TLR4 has been shown to recognize chlamydial heat shock protein [28], [29], respiratory syncytial virus fusion (F) protein [30] and molecular patterns of malarial parasites [31]. Two functional mutations in the extracellular LRR domain of human *TLR4* have been identified at position A896C, leading to A/G amino acid transition at position 299, and C1196T, leading to T/I transition at position 399 [13], [28]. Although co-segregation of the D299G and T399I genotype occurs approximately 98% of Western populations, both polymorphic variants appeared have been shown to independently affect responsiveness to inhaled LPS. Recent stoichiometric analysis of the D299G and T399I polymorphic variants of TLR4 confirmed that the presence of each of the D299G and T399I variants results in distinct structural changes in the ligand-binding site of the receptor [32], which account for increased susceptibility to infectious disease such as Gram-negative sepsis [9] and malaria [31], [33].

As TLR4 mutations have been shown to determine susceptibility to a number of exogenous infectious agents, should we consider potential infectious agents responsible for causing preeclampsia or HELLP syndrome? Recently, it has indeed been demonstrated that women with preeclampsia are prone to periodontal infections, mainly caused by gram-negative bacteria [34]. Other associations with preeclampsia have been observed for adeno-associated virus 2 (AAV-2) [35], parvovirus B19 [36] and cytomegalovirus [37]. Of interest, preeclampsia appears more commonly in women who are seropositive for *Chlamydia Pneumoniae* [38], which is a TLR4 dependent intracellular pathogen related to the development of atheromatous lesions. Also, in a recent study of 304 pregnant women infected with *P. Falciparum* from South Ghana, the D299G allelic variant of TLR4 was associated with a 5-fold increased risk of severe maternal anemia, as a result of ineffective clearance of the malaria parasites [39]. Although the interaction of TLR4 with *P. Falciparum* is not fully understood, Krishnegowda and colleagues showed in vitro that TLR4 can be activated by malarial glycosylphosphatidylinositols (GPIs) to induce a pro-inflammatory response. It is, however, not known if the D299G variant has any effect on this interaction [31]. Furthermore, in women with HELLP syndrome, in vitro experiments show striking differences in monocyte handling of LPS and gram-negative bacteria, when compared to women with preeclampsia only [40]. In our study, TLR4 mutations were 2.4 times more common in women with HELLP syndrome than those with preeclampsia only, suggesting that the TLR4 pathway might be involved in the development of HELLP episodes in women with early-onset preeclampsia. The HELLP syndrome is generally considered the clinical extreme of preeclampsia, although its acute onset and frequent spontaneous (albeit temporary) resolution within days, even in those women who do not deliver [41], [42], suggests a more rapid deterioration initiated by temporally released, yet unknown triggers. Our data suggest that exogenous (infectious) agents involved in the TLR4 pathway, might be candidate factors triggering HELLP syndrome within the already immuno-modulated pre-eclamptic patient. On the other hand, it has been suggested that TLR4 can be activated by number of endogenous ligands involved in vascular damage, including heat-shock protein 60 and the extracellular domain A (EDA) of fibronectin [43], [44]. Although these data are inconclusive because of potential LPS contamination during analytical procedures, they suggest a role for TLR4 in the so-called “danger hypothesis” [45]. This hypothesis states that tissue damage (either by endogenous or exogenous agents) involves the release of signalling molecules that are capable of eliciting an immediate immune reaction. In preeclampsia, elevated circulating levels of cellular and sub-cellular placental debris, including shedding of syncytiotrophoblast membrane fragments [1], [3], cell-free fetal DNA [46] and soluble fms-like tyrosine kinase 1 (sFlt-1) [47], have been shown to induce endothelial damage and precede onset of the clinical syndrome. Interestingly, recent data by Kim and colleagues showed increased expression of TLR4 in interstitial trophoblasts in placental bed biopsies obtained from women with preterm preeclampsia, consistent with a role for TLR4 at the feto-maternal interface [48]. However, further studies are needed to investigate the possibility that TLR4 acts as a danger receptor involved in clearance of excess shedding of placental factors.

NOD2 is a member of the Apaf1/Ced4 superfamily of apoptosis regulators, mainly expressed in monocytes, that acts as an intracellular pattern recognition proteins (PRRs), containing LRR domains similar to TLR4 [14]. Although NOD proteins have an indirect role in LPS signaling, NOD2 primarily recognizes a muramyl dipeptide (MDP) motif of peptiglycans found in virtually all classes of bacteria, similar to the NOD1 ligand diaminopimelic acid (DAP)-containing muramyl tripeptide (M-TriDAP), found in gram-negative bacteria. In addition to its role as an intracellular PRR, genetic mapping studies have identified NOD2 as a strong susceptibility gene for Crohn's disease [14], [15]. The allelic variants L1007fs, R702W and G908R, each represent three independent haplotypes commonly observed in patients with European ancestry. Individuals homozygous for any of these mutations exhibit an odds ratio of 42.1 for developing Crohn's disease. Recent studies have clarified the genetic and molecular basis for this association, as reviewed in reference [14]. All three variants affect the LRR-domain and are considered loss-of-function mutations, likely to impair the recognition of microbial components, interfering with appropriate activation of NF-κB in monocytes, as well as disregulating NOD1 function. Also, NOD2-/- mice exhibit a TH-1 type pro-inflammatory phenotype following stimulation with TLR agonists [49], that suggests a more fundamental role for NOD2 in controlling the innate immune response. Although the role of NOD proteins in pregnancy and placentation has not yet been investigated, protein expression and induction of pro-inflammatory cytokine release after NOD1 and NOD2 activation, was recently observed in first trimester trophoblast cells [50]. In our study, no association was observed for common NOD2 polymorphisms alone and a history of early-onset preeclampsia or HELLP syndrome. This is in contrast to one previous study by Goddard et al [51]. In this study, a significant association was observed with NOD2 polymorphisms in women with preeclampsia, mostly late-onset disease, by a global haplotype-based approach. However, our data do suggest a combined association of NOD2 allelic variants and maternal pro-inflammatory phenotype with pregnancy outcome, which will be further discussed below.

In addition, we observed elevated levels of circulating biomarkers for inflammation and the acute-phase response in women with a history of early-onset preeclampsia, when measured at least six months after delivery, including CRP, fibrinogen and IL-6, as compared to women who experienced only uneventful pregnancies. Similar to previous reports [27], our data demonstrate that non-pregnant women with a history of early-onset preeclampsia more frequently exhibit a pro-inflammatory phenotype. Biomarkers of the acute-phase inflammatory response, including CRP, fibrinogen, IL-6 and vWf, have all been shown to be predictive of long-term cardiovascular risk, although not independently adding to classic risk factor algorithms [52]. This study suggests that an innate maternal pro-inflammatory phenotype might be a common underlying risk factor for early-onset preeclampsia and cardiovascular disease [53]. Surprisingly, we observed an inverse relationship between sICAM-1 levels and history of early-onset preeclampsia, which is in contrast with previous findings by Freeman and colleagues [27]. However, their study reported on sICAM-1 levels determined 20 years after delivery, whereas our data represent samples collected at a mean interval between delivery and enrolment of 0.7 years (range 0.5 to 8.5 years).

In this study, elevated plasma inflammatory biomarker levels were unaffected by TLR4 and NOD2 genotype, and correlated with increasing odds ratios for early-onset preeclampsia even after careful correction for confounding factors, including age, interval between delivery and enrolment, body-mass index, smoking status and chronic hypertension. When examining the combined association of TLR4 or NOD2 mutations and circulating levels of inflammatory markers, highest odds ratios were observed for women carrying allelic variants of TLR4 or NOD2, who were in the upper tertiles of CRP, fibrinogen and IL-6. Compared to women with plasma levels within the lowest tertiles of the control distribution and negative for any of the TLR4 and NOD2 polymorphisms, the highest tertiles of fibrinogen and IL-6 and the presence of attenuating allelic variants of TLR4 or NOD2 showed both independent and combined associations with a history of early-onset preeclampsia.

Our case-control study has several strengths and limitations. To our knowledge, this study describes genotype and phenotype data of the largest series of women with a history of early-onset preeclampsia and HELLP syndrome yet, recruited over an interval of more than a decade. However, we designed our study to include only primiparous women, who delivered before 34 weeks of gestation. Therefore, our data may not be extrapolated to multiparae and women with a history of late-onset or near term preeclampsia, as the pathophysiology of the disorder leading to onset after 34 weeks' gestation might differ from that which causes early-onset disease [21]. A major strength of our study is in the assessment of genetic single-nucleotide polymorphisms, which unlike acquired or environmental risk factors are fixed at birth and therefore provide a measure of life-long exposure, not likely to be subject to confounding due to the timing of investigation [54]. Despite this considerable advantage, in any genetic association study with a case-control design, selection bias and population stratification should be considered. Conversely, because plasma samples for analysis of inflammatory biomarkers were collected after pregnancy, we cannot exclude the possibility that pregnancy itself might have led to temporary or permanent changes in inflammatory status. For some (IL-6 and sICAM-1) but not all (CRP, fibrinogen and vWf) of the measured inflammatory markers we indeed observed a significant correlation with the interval between delivery and enrolment, suggestive of at least short-term changes up to several months after pregnancy. Nonetheless, the observed associations between high levels of interleukin-6 and fibrinogen and a positive history of early-onset preeclampsia were only moderately influenced by adjustment for interval between delivery and sampling. At present no prospective data comparing pre- and post-pregnancy levels of inflammatory markers are available to provide a more conclusive answer to this question.

In summary, we are the first to show a relationship between allelic variants of TLR4, that impair the innate inflammatory response to LPS, early-onset preeclampsia and HELLP syndrome. Next, we observed that a history of early-onset preeclampsia is related to higher plasma levels of CRP, fibrinogen and IL-6 at least six months after delivery. Our results support the hypothesis that the innate immune system contributes to maternal susceptibility to early-onset preeclampsia and HELLP syndrome. Therefore, identification of components of the maternal innate inflammatory system that predispose to hypertensive disorders of pregnancy merits further investigation.
